# A scoping review of photovoice studies concerning greenspaces and their impacts on health: Protocol

**DOI:** 10.1371/journal.pone.0355059

**Published:** 2026-08-04

**Authors:** Chidera M. Ejike, Rebecca Ramos, Christiana Ketema, Mackenzie Waller, Kai Glahn, Auwal Abubakar, Deborah Jean McClelland, Kacey Ernst, Huaqing Wang, Shujuan Li, Maiya Grace Block Ngaybe

**Affiliations:** 1 Department of Epidemiology and Biostatistics, The University of Arizona, Tucson, Arizona, United States of America; 2 Department of Community, Environment, and Policy, The University of Arizona, Tucson, Arizona, United States of America; 3 Mel and Enid Zuckerman College of Public Health, The University of Arizona, Tucson, Arizona, United States of America; 4 College of Architectural Planning and Landscape Architecture, The University of Arizona, Tucson, Arizona, United States of America; 5 Department of Psychiatry, The University of Arizona, Tucson, Arizona, United States of America; 6 The University of Arizona, Tucson, Arizona, United States of America; 7 Arizona Health Sciences Library, The University of Arizona, Tucson, Arizona, United States of America; 8 Department of Landscape Architecture and Environmental Planning, Utah State University, Logan, Utah, United States of America; The University of Iowa College of Public Health, UNITED STATES OF AMERICA

## Abstract

**Objective:**

The objective of this scoping review is to characterize the extent and nature of the available research on photovoice studies examining how greenspaces influence community health, and to identify community actions and advocacy arising from these participatory projects.

**Introduction:**

Greenspaces have been shown to support physical, mental, and social health. Yet research often overlooks community perspectives, particularly in arid regions. Photovoice enables individuals to document and reflect on their lived environments, providing unique insights into the relationships between greenspaces, health, and community wellbeing. Synthesizing photovoice studies that examine how greenspaces impact health across different ecosystems and landscape designs is essential to capture community-driven knowledge and outcomes, including initiatives that inform policy and practice. The protocol is registered in Open Science Framework (https://doi.org/10.17605/OSF.IO/2DYGA).

**Inclusion criteria:**

This review will include primary studies employing photovoice methods with human participants, of any demographic or cultural background, that focus on greenspaces and associated health outcomes. Eligible studies may use qualitative or mixed methods designs, provided photovoice is a core component. Quantitative-only studies, reviews, editorials, and those addressing distal health measures will be excluded.

**Methods:**

Searches will be conducted in electronic databases (PubMed, PsycINFO, Embase, ERIC, CINAHL, Web of Science) and relevant grey literature sources. No language restrictions will be applied, and the time frame will extend from January 1997, when photovoice was first described as a methodology, to the present. Non-English studies will be translated using available translation tools and/or reviewed by a multilingual member of the team or colleague where feasible. Screening and data extraction will be conducted in duplicate using Covidence. Findings will be summarized descriptively and synthesized thematically. Empirical studies will be appraised using the JBI Critical Appraisal Tools, while grey literature source quality will be assessed using the AACODS checklist. Confidence in the synthesized qualitative findings will be assessed using GRADE-CERQual (Confidence in the Evidence from Reviews of Qualitative Research).

## Introduction

Greenspace is generally understood as land that is covered with vegetation, including grass, trees, and shrubs. It plays a vital role in urban and rural environments. In city settings, these spaces include parks, gardens, tree-lined streets, planted lots, and urban forests that are often publicly accessible [1]. Extensive research has demonstrated that exposure to greenspace offer numerous benefits to human health, including physical, mental, social, and emotional well-being [2,3]. Exposure to greenspaces is associated with reductions in stress, improvements in cardiovascular health, increases in physical activity, and enhancements in community cohesion and social capital [4,5]. However, much of the existing literature focuses on quantitative outcomes and often overlooks community members’ lived experiences and perceptions of how greenspaces impact their health. Participatory research methods, such as photovoice, offer a unique avenue for exploring and understanding community perspectives on the relationship between greenspaces and health [6].

There is a growing academic and policy interest in understanding how greenspaces impact health outcomes. Yet, despite this interest, there is a notable gap in synthesizing evidence, specifically from photovoice studies, that captures community voices and actions related to greenspace and health. These participatory studies often reveal nuanced influences of greenspace quality, accessibility, cultural significance, and environmental justice aspects on perceived health benefits or harms [7]. Furthermore, greenspace and health research has been unevenly distributed across geographic, climatic, and socioeconomic contexts.^8^ In this review, “underrepresented environments” refers to settings that have received comparatively less attention in the greenspace and health literature, including arid and semi-arid regions, tropical regions, rural and peri-urban communities, and low- and middle-income countries (LMICs). These contexts may experience different greenspace conditions, including variation in vegetation type, water availability, maintenance needs, cultural uses of outdoor space, heat exposure, and infrastructure investment. As a result, community experiences of greenspace access, quality, safety, and health benefits may differ substantially from those reported in more commonly studied urban and temperate high-income settings.

Different environmental and geographic contexts present distinct challenges and opportunities regarding greenspace characteristics and their relationship to health [7,8]. For example, greenspaces in arid and semi-arid environments may differ from temperate settings in vegetation, water and shade availability, heat exposure, and community preferences for drought-tolerant or native landscapes. Similarly, greenspaces in tropical, rural, peri-urban, and LMIC settings may reflect different patterns of land use, maintenance, access, cultural meaning, environmental risk, and public health need. Arid greenspaces often require resource adaptations, such as prioritizing drought-tolerant native plants, and may face competing demands between ecological sustainability and community preferences for lush vegetation [8,9]. However, evidence describing how these landscape characteristics influence health and well-being, particularly from the perspectives of residents in underrepresented environments, remains sparse and fragmented across disciplines and study designs [8–10]. Research often overlooks community perspectives, particularly in underrepresented environmental and geographic contexts, including arid and semi-arid regions, tropical regions, rural communities, and low- and middle-income countries. These contexts may differ substantially in greenspace availability, vegetation type, climate-related constraints, cultural meanings of greenspace, and community priorities for health and wellbeing. A comparative understanding of greenspace experiences across diverse environmental contexts can help identify and tailor equitable and ecologically appropriate strategies for public health, urban planning, and community-engaged greenspace interventions [11].

Studies examining how greenspaces impact community health are more prevalent in High Income Countries (HICs), such as the Netherlands and the United States [12]. However, there remains a need for studies in LMICs. Therefore, examining where this research is conducted globally is important for identifying gaps and understanding differences in cultural contexts, communities’ lived experiences, and local priorities [12,13].

A scoping review design was selected as the most appropriate methodology for several reasons. Unlike systematic reviews, which focus on more specific questions and often assess the effectiveness of interventions, scoping reviews are ideal for mapping broader, more complex, and emerging fields of evidence [14]. This review’s objective to explore participatory methodologies, diverse health outcomes, and various environmental contexts aligns well with the exploratory and descriptive nature of scoping reviews. Additionally, as photovoice studies employ heterogeneous methods and report a wide range of qualitative data, a scoping review can comprehensively capture this variation, laying the groundwork for future targeted systematic assessments or qualitative syntheses [15]. A preliminary search of MEDLINE, the Cochrane Database of Systematic Reviews, Open Science Framework, and the JBI Evidence Synthesis database did not identify any current or ongoing scoping reviews specifically addressing photovoice studies on greenspaces and their impacts on community health. This review will therefore fill a critical gap by clarifying how different types of greenspaces are valued and utilized across diverse settings, illuminating the breadth of health outcomes identified by communities, and providing an organized synthesis accessible to researchers, policymakers, public health practitioners, and community stakeholders aiming to leverage community-engaged research for health-promoting greenspace interventions.

The objective of this scoping review is to map and synthesize the available evidence on photovoice studies examining the impacts of greenspaces on community health and community actions that arise from these participatory projects, with particular attention to environmental context differences, including arid and non-arid settings. This review aims to address the following gaps: (1) the range and types of greenspaces studied across diverse environmental contexts; (2) the health outcomes identified through community perspectives; and (3) the community-driven advocacy, policy influence, or planning that emerges as a result of photovoice research. This review will adhere to the Johanna Briggs Institute (JBI) methodology [16] to ensure comprehensive and transparent reporting of findings. The protocol is registered in Open Science Framework (https://doi.org/10.17605/OSF.IO/2DYGA).

## Review questions

### Population

- What are the characteristics of the communities participating in photovoice greenspace studies (e.g., geographic location, socio-demographics, etc.)?

### Concepts

- How are photovoice studies used to examine the relationship between greenspaces and community health?
- What health outcomes related to greenspace exposure are identified in photovoice studies?
- What forms of community action or advocacy emerge from photovoice studies on greenspaces and health?
- How does the photovoice methodology offer a unique perspective and contribution to the greenspace-health literature?
- What types of greenspaces are examined in photovoice studies?
- How is greenspace defined across photovoice studies?
- What ethical considerations are addressed in greenspace and health photovoice studies?

### Context

- Do community perceptions of greenspace and health differ across environmental and geographic contexts, including arid, semi-arid, temperate, tropical, urban, peri-urban, rural, and low- and middle-income country settings?
- In which geographic, climatic, and environmental contexts are photovoice greenspace and health studies being conducted?
- How do contextual differences, including climate, urbanicity, vegetation type, infrastructure, culture, and resource availability, influence perceived health outcomes related to greenspaces?

## Inclusion criteria

### Participants

This review will include studies involving human participants of any age, gender, socioeconomic status, and/or cultural background. Eligible participants may be individuals, groups, or communities engaged in photovoice projects that explore greenspaces and health. No restrictions will be applied regarding geographic location, setting (urban, peri-urban, or rural), or population type. This broad scope is intended to capture evidence from contexts that are less frequently represented in greenspace and health research, including low- and middle-income countries, rural and peri-urban communities, tropical regions, and arid or semi-arid environments, where greenspace availability, form, function, and community meaning may differ from more commonly studied urban and temperate high-income settings. Studies will be excluded if participants are not directly engaged in photovoice activities (e.g., projects in which photographs are taken or analyzed solely by researchers without community involvement), unless the term photovoice was explicitly mentioned as the methodology used in such studies.

### Concept

The central concept of this scoping review is the application of the photovoice methodology to investigate how community members perceive and experience greenspaces and their impacts on health and well-being. Photovoice is a participatory research approach in which community members use photography, combined with critical dialogue, to document, reflect on, and communicate their lived experiences within their environment [6]. Eligible studies must explicitly employ photovoice as a core design, where participants take photographs reflecting their lived experiences and engage in participatory analysis and dissemination with researchers. Quantitative-only studies, reviews, editorials, and other non-original research will be excluded, while mixed-methods studies will be included only when photovoice is a distinct, reported component. For the purposes of this review, studies must clearly investigate greenspaces as defined in the introductory section; those that broadly address the “built environment” without distinct exposure to greenspace will be excluded unless greenspace findings can be extracted and analyzed separately. Health outcomes of interest will include direct, individual-level impacts of greenspace exposure, such as physical health (e.g., cardiovascular health), mental health (e.g., stress reduction, mental wellbeing), social implications (e.g., community cohesion), and emotional outcomes (e.g., improved mood). Studies that focus exclusively on distal or systemic outcomes (e.g., social determinants of health or policy discussions without direct measurement of health outcomes) will be excluded. Where available, this review will also chart the differences in health impacts across multiple types of greenspaces, including managed environments (e.g., maintained parks, golf courses) and naturalistic or native-plant landscapes.

### Context

This review will consider studies conducted in any geographic or cultural setting where photovoice has been applied to explore greenspaces and health. Eligible contexts include urban, peri-urban, and rural environments, across diverse climates, including arid, semi-arid, temperate, and tropical regions. Studies may be situated in community, educational, residential, or clinical settings, provided the focus remains on greenspaces as defined in the Concept section. No restrictions will be placed on country, region, or income level, ensuring global perspectives are captured. Similarly, there will be no language restrictions; studies published in languages other than English will be included if sufficient information can be obtained through translation. Non-English titles and abstracts will first be translated using available translation tools and, when needed, reviewed by multilingual members of the team or qualified colleagues. For studies that appear eligible at the full-text stage, translation will be conducted using either an interpreter or employ machine translation, possibly Whisper AI, with human verification. If translation to English cannot be obtained to determine eligibility or extract relevant data, the source will be documented and excluded with the reason noted. Exclusions will apply to studies examining environments not characterized as greenspaces (e.g., purely built environments without vegetation). Studies conducted exclusively in virtual or simulated environments will also be excluded.

## Included sources

This scoping review will primarily consider qualitative studies, as photovoice is a participatory qualitative methodology. We will include primary research articles in which photovoice is the main methodology or is part of a mixed-methods study. Quantitative-only studies, narrative reviews, editorials, commentaries, and opinion papers without a distinct photovoice component will be excluded. In addition, systematic reviews will be excluded unless they contain primary data not otherwise available in published studies. Grey literature sources such as dissertations, theses, community or organizational reports, and other non–peer-reviewed outputs will be considered where they report original photovoice findings.

## Methods

The proposed scoping review will be conducted in accordance with the JBI methodology including the reporting guidelines set out by the PRISMA-ScR (Preferred Reporting Items for Systematic reviews and Meta-Analyses extension for Scoping Reviews) and PRISMA-S extensions [16,17].

### Search strategy

The search strategy aims to identify both published and unpublished studies on photovoice methodology, greenspace exposure and health outcomes. A three-step search approach will be used in this review. Firstly, a search strategy which utilized keywords identified in the titles and abstracts of relevant articles has been developed for PubMed (National Library of Medicine), with the addition of index terms (MeSH). This search strategy will be translated to other electronic databases:, PsycINFO (EBSCOhost), ERIC, CINAHL (EBSCOhost), Embase (Elsevier), and Web of Science Core Collection (Clarivate). (see Appendix 1 (S1 File)). When possible, the human filter will be applied.

Studies published in any language will be included to reduce language bias and ensure representation of evidence from underrepresented geographic and socioeconomic contexts worldwide. Non-English titles and abstracts will initially be translated using available translation tools. Where necessary and feasible, translations will be reviewed by multilingual members of the review team or qualified colleagues. Potentially eligible full-text articles will undergo machine translation with human verification whenever feasible. If an adequate translation cannot be obtained, the study will be documented and excluded, with the reason reported. Studies published from 1997 onward will be included, as the original articles describing the photovoice methodology appeared in the late 1990s. The PubMed search strategy was developed with input from the project team and refined in consultation with an experienced health sciences librarian familiar with scoping review methodology. The PubMed search strategy will be translated to the other databases, taking into account their controlled vocabularies and syntax. Snowball sampling techniques, including screening reference lists of included studies and key reviews, will be used to identify additional relevant sources. ProQuest Dissertations and Theses Global will be searched to include grey literature and unpublished studies. Google Scholar will also finally be searched to identify possible additional peer-reviewed literature relevant to the study objectives.

## Studies/source of evidence selection

After the search of the electronic databases is complete, all identified citations will be exported from the databases, compiled and uploaded to Covidence, a web-based software platform designed to streamline and manage the process of conducting systematic reviews, and duplicates will be eliminated. Titles and abstracts will be screened by two or more independent reviewers to evaluate compliance with the review's inclusion criteria. Reasons for excluding evidence sources at the full-text level that do not meet the inclusion criteria will be recorded and reported in the scoping review. Any disagreements between reviewers at each stage of the selection process will be resolved through discussion or by involving additional reviewers to reach a consensus. The search results and the study inclusion process will be fully reported in the final scoping review, presented in a PRISMA (Preferred Reporting Items for Systematic reviews and Meta-Analyses) flow diagram, and documented according to PRISMA-ScR (Preferred Reporting Items for Systematic reviews and Meta-Analyses extension for Scoping Reviews) [17].

## Data extraction

Data will be extracted by two or more independent reviewers using a data extraction tool developed by the reviewers. Extracted data will include participant details, the concept, the context, the study methods, and key findings relevant to the review question(s). A draft extraction form is provided (see Appendix 2 (S1 File)). The draft data extraction tool will be revised iteratively during the data extraction process for each included evidence source. Main outcomes extracted will be physical, mental, and social/community health as they are most central to the review questions, while additional outcomes will include environmental, behavioral, advocacy, policy, access, and other emergent community-defined impacts to capture the full scope of evidence. Modifications will be detailed in the scoping review. Any disagreements between the reviewers will be resolved through discussion or by involving additional reviewers. If appropriate, authors of papers will be contacted to request missing or additional data, where required.

## Critical appraisal of individual sources of evidence

To provide additional context regarding the methodological rigor of the included photovoice studies, a quality assessment will be performed using the Johanna Briggs Institute (JBI) Critical Appraisal Tools. The JBI critical appraisal tools are well-suited for evaluating qualitative research, focusing on study validity, methodology, ethical considerations, data analysis, and relevance of findings [16]. The AACODS checklist (Authority, Accuracy, Coverage, Objectivity, Date, and Significance) will additionally be used to assess the quality of grey literature sources [18]. This appraisal will not be used to exclude studies but rather to inform the interpretation and synthesis of evidence, identify gaps related to study quality, and guide recommendations for future research. Two independent reviewers will independently complete the appropriate JBI Critical Appraisal Tool for eligible studies and the AACODS checklist for grey literature sources, with any disagreements resolved through discussion or consultation with a third reviewer when necessary.

## Data analysis and presentation

The evidence gathered from this review will be systematically organized and presented to directly address the review objective and research questions. Descriptive data (such as study characteristics, participant demographics, and types of greenspaces studied) will be summarized in tabular form to provide a clear overview of the existing literature. Qualitative findings (including community perceptions of greenspaces, reported health outcomes, and community-driven actions) will be synthesized thematically. Mapping techniques such as conceptual diagrams or frameworks may be used to visually represent relationships among photovoice methodologies, greenspace contexts, and health impacts identified across studies. A narrative summary will accompany all tabulated and/or graphical data, provide interpretative context, and explicitly link the results to the review’s objectives and specific review questions. This approach will support the identification of research gaps, variations by geographic or environmental context, and implications for future participatory research. Confidence in the synthesized qualitative review findings will be assessed using the Confidence in the Evidence from Reviews of Qualitative Research (GRADE-CERQual) approach for qualitative studies [19]. For mixed-methods or descriptive findings that do not fit GRADE-CERQual, we will summarize the strength and limitations of the evidence narratively, informed by the JBI Critical Appraisal Tools and the AACODS checklist for grey literature.

## Supporting information

S1 File Appendices. Appendix 1: PubMed search strategy and Appendix 2: Data extraction instrument.
(DOCX)

S2 File PRISMA-P checklist. PRISMA-P (Preferred Reporting Items for Systematic review and Meta-Analysis Protocols) 2015 checklist.
(DOCX)
