# Correction: A scoping review of photovoice studies concerning greenspaces and their impacts on health: Protocol

**DOI:** 10.1371/journal.pone.0361454

**Published:** 2026-10-23

**Authors:** Chidera M. Ejike, Rebecca Ramos, Christiana Ketema, Mackenzie Waller, Kai Glahn, Auwal Abubakar, Deborah Jean McClelland, Kacey Ernst, Huaqing Wang, Shujuan Li, Maiya Grace Block Ngaybe

## Notice of Republication

An incomplete, earlier version of this article was published in error, for which the publisher apologizes. This article was republished on October 12^th^, 2026 to correct this. Please download the article again to view the correct version. The originally published, uncorrected article and the republished, corrected article are provided here for reference.

## Supporting information

S1 File Originally published, uncorrected article. (PDF)

S2 File Republished, corrected article. (PDF)
