# Supplementary material for: A scoping review of photovoice studies concerning greenspaces and their impacts on health: Protocol
Source: PLoS One. 2026 Aug 4;21(8):e0355059. doi: 10.1371/journal.pone.0355059 (PMC13436740; doi:10.1371/journal.pone.0355059)
Supplement: Supplementary file 1 [file pone.0355059.s001.docx]

**Appendices**

**Appendix 1:** PubMed Search Strategy

|  | **Greenspace Terms** | **Photovoice Terms** | **Health Terms** |
| --- | --- | --- | --- |
| **MeSH Terms** | “gardening”[MeSH] OR “gardens”[MeSH] OR “nature”[MeSH] OR “trees”[MeSH] OR “forests”[MeSH] OR |  | “health”[MeSH] OR |
| **Keywords** | (Greenspace OR "Green space" OR Garden OR "Urban forest" OR Nature OR Park OR Parkland OR "Urban habitat” OR "Urban green" OR "Urban ecosystem" OR "Urban trees" OR Tree OR Woodland OR Landscape OR "Vegetated area" OR "Green environment" OR "Green element" OR "Green network" OR "Green corridor" OR "Green infrastructure" OR "Greening project" OR "Green belt" OR Greenbelt OR "Green roof" OR Greenery OR "Natural environment" OR "Urban greenery" OR Greenness) AND | (photovoice OR “photo novella” OR photonovella OR Photonovela OR fotonovela OR “photo-elicitation") AND | (Health OR Wellbeing OR “Well-being” OR Wellness) |
| **Hits** | **388635** | **2318** | **8163253** |

Combined = 319; Limited Dates to 1997-2026

**Appendix 2: Data extraction instrument**

| **Category** | **Extraction Item** |
| --- | --- |
| Bibliographic Information | Author(s) |
|  | Year of publication |
|  | Title of study |
|  | Journal/Source |
|  | DOI/URL |
| Study Characteristics | Study design (qualitative, mixed methods, CBPR, etc.) |
|  | Methodological approach (photovoice, PAR, etc.) |
|  | Study duration |
|  | Funding source (if noted) |
| Community Characteristics | Location (country, region, city/town, urban/rural, arid/non-arid) |
|  | Population (age groups, gender, socio-demographics, marginalized groups) |
|  | Community size/sample size |
| Concepts & Application of Photovoice | Purpose of photovoice (exploration, advocacy, evaluation, etc.) |
|  | How photovoice was conducted (participants, process steps, engagement level) |
|  | Forms of community action/advocacy resulting from photovoice |
| Greenspace Characteristics | Type(s) of greenspace studied (parks, gardens, urban forests, etc.) |
|  | Definition of greenspace used (author/community definition) |
| Health Impacts & Outcomes | Physical health outcomes (e.g., activity, chronic disease prevention) |
|  | Mental health outcomes (e.g., stress reduction, well-being) |
|  | Social/community outcomes (e.g., cohesion, resilience, empowerment) |
|  | Other outcomes noted |
| Ethical Considerations | Consent and photo ownership |
|  | Confidentiality considerations |
|  | Power dynamics/community agency issues |
|  | Other ethical concerns |
| Contextual Factors | Environmental context (arid vs. non-arid, climate, seasonality) |
|  | Geographic context (urban, suburban, rural) |
|  | Reported contextual influences on greenspace-health relationships |
| Key Findings & Implications | Summary of main findings |
|  | Implications for practice or policy |
|  | Gaps identified by authors |
| Reviewer Notes | Relevance to review questions |
|  | Strengths/limitations of the study |
|  | Additional comments |
