# Supplementary material for: A scoping review of photovoice studies concerning greenspaces and their impacts on health: Protocol
Source: PLoS One. 2026 Aug 4;21(8):e0355059. doi: 10.1371/journal.pone.0355059 (PMC13436740; doi:10.1371/journal.pone.0355059)
Supplement: Supplementary file 3 [file pone.0355059.s003.docx]

**A Scoping Review of Photovoice Studies Concerning Greenspaces and Their Impacts on Health: Protocol**

1. **Summary of the research and my overall impression**

This research protocol outlines a scoping review designed to map and synthesize evidence from photovoice studies that examine how greenspaces influence community health. The authors provide a well-articulated rationale, noting that while the health benefits of greenspaces are well established, existing literature predominantly relies on quantitative approaches and largely overlooks community-lived experiences-particularly in underrepresented settings such as arid regions and Low- and Middle-Income Countries (LMICs).

The methodology for this scoping review will follow the JBI methodology and adopt a broad, inclusive approach with no restrictions on language, geography, or demographic background. Eligible sources include qualitative and mixed-methods primary studies in which photovoice is a core component, and these source publications that will be considered must not be earlier than 1997 (when photovoice was first described) up till this present time. Searches will be carried out across nine major electronic databases, including PubMed, CINAHL, Embase, and PsycINFO, supplemented by grey literature sources such as ProQuest Dissertations and Google Scholar. Screening and data extraction will be conducted in duplicate using Covidence, with disagreements resolved by consensus or the engagement of a third reviewer.

Data will be synthesized descriptively and thematically organized around three main domains: the types and characteristics of greenspaces studied, health outcomes identified through community perspectives, and community-driven advocacy or policy actions arising from the photovoice process. The quality of the outputs will be appraised using the JBI Critical Appraisal Tools, not for exclusion purposes but to contextualize findings. The GRADE approach will be used to assess the overall strength of evidence gathered. The review is currently ongoing, with record screening in progress and final results anticipated by May 2027.

Overall, this is a timely, well thought-out, and methodologically sound protocol that addresses a genuine and clearly articulated gap in the participatory research literature. The decision to use a scoping review design is appropriate and well-justified, given the heterogeneous nature of photovoice studies and the exploratory intent of this review. The authors demonstrate a sound knowledge of both photovoice as a methodology and the broader greenspace-health evidence base.

The protocol's strengths include its global and inclusive scope, its deliberate attention to equity-particularly the focus on arid environments and LMICs -and its adherence to transparent, reproducible JBI and PRISMA-ScR17 reporting standards. The preliminary database search that was conducted to confirm the non-existence of any similar reviews further strengthens the case for this great work.

**2. Some specific areas for improvement**

| Line (s) | Issue (s) | Comment | Adjustment required |
| --- | --- | --- | --- |
| Major issues | | | |
| 27-28 | ‘… particularly in underrepresented environments such as arid regions’ | 1. This statement creates some ambiguity to me. What is the definition of underrepresented environments? 2. Regarding the examples of the underrepresented environments’ given in the protocol, arid was more spoken about more whereas as stated in line 132-134 that ‘No restrictions will be applied regarding geographic location, setting (urban, peri-urban, or rural), or population’. The way the document is written (like in line 63), presents review as majorly concerned with arid regions but not the other regions that fall under tropical, temperate, continental or polar climate. | 1. Please define what under-represented environment means. 2. You may need to mention other regions as outlined in the main protocol like the LMICs mentioned, as well as include some information around what is obtainable there regarding the subject matter. |
| 38 | ‘Searches will be conducted in electronic databases and relevant grey literature sources’. | 1. Since grey literature documents are rarely peer-reviewed how will you assess their quality and reliability? What tool will you be using to evaluate the Authority, Accuracy, Coverage, Objectivity, Date, and Significance of the grey literatures? 2. The decision to limit the broad Google search to the first 200 hits (line 202), although pragmatic, may benefit from a brief justification, as this threshold could miss relevant community or organizational reports. | 1. State what tool you will use to evaluate the grey literatures that will be assessed aside the JBI Critical Assessment tools that you would be using to evaluate the findings. 2. How would you manage the issue around using the method of the first 200 hits? |
| 39 | ‘No language restrictions will be applied’ | Any plan for translation? The protocol does not specify how non-English studies will be translated or assessed for quality, which is a practical gap worth addressing. | As stated in line 165, there is a need to state how you would manage issues with findings that are not presented in English, and how the quality of the translations. This should also be mentioned briefly in the abstract. |
| 165 | ‘Similarly, there will be no language restrictions; studies published in languages other than English will be included if sufficient information can be obtained through translation’. |  |  |
| 248 | ‘The strength of the body of evidence will be assessed using the GRADE 248 (Grading of Recommendations, Assessment, Development, and Evaluations) approach’. | The use of GRADE, typically designed for intervention-based evidence, especially in healthcare is noted. | Please clarify how you intend to adapt or apply GRADE appropriately to predominantly qualitative, and participatory evidences that would be gathered. |
| Minor issue (s) | | | |
| 326-328 | Reference 17 in the reference section seems missing in the entire body of the document |  | Please review the references and adjust as required. |

3. **Recommendations**

In all, this is a strong and publication-ready protocol with minor revisions recommended. The work has the potential to make a meaningful contribution to participatory environmental health research, particularly for communities and policymakers seeking evidence-based, community-informed greenspace interventions.
