# Supplementary material for: A scoping review of photovoice studies concerning greenspaces and their impacts on health: Protocol
Source: PLoS One. 2026 Aug 4;21(8):e0355059. doi: 10.1371/journal.pone.0355059 (PMC13436740; doi:10.1371/journal.pone.0355059)
Supplement: Supplementary file 4 [file pone.0355059.s005.docx]

July 13, 2026

Dear Editor,

Thank you for the opportunity to revise our manuscript, "A Scoping Review of Photovoice Studies Concerning Greenspaces and Their Impacts on Health: Protocol," and for the reviewer’s thoughtful and constructive feedback. We have carefully considered each comment and revised the manuscript to improve clarity, methodological transparency, and alignment across the abstract, introduction, inclusion criteria, search strategy, critical appraisal, and data analysis sections.

The table below provides a point-by-point response to each comment, followed by a table of additional changes made by the author team to further strengthen the manuscript. Line numbers refer to the originally submitted manuscript; all revisions are shown in the tracked-changes version.

**Responses to Reviewer Comments**

| **Reviewer comment** | **Response** |
| --- | --- |
| **Lines 27–28 (major)**  *Please define what under-represented environment means. You may need to mention other regions as outlined in the main protocol like the LMICs mentioned, as well as include some information around what is obtainable there regarding the subject matter.* | Thank you for this important clarification. The Introduction now explicitly defines "underrepresented environments" as settings that have received comparatively less attention in the greenspace and health literature, including arid and semi-arid regions, tropical regions, rural and peri-urban communities, and low- and middle-income countries (LMICs). Arid regions are now presented as one example rather than the primary focus, and the abstract was revised accordingly.  We also added text describing what is obtainable in these settings: differences in vegetation type, water and shade availability, maintenance needs, cultural uses of outdoor space, heat exposure, and infrastructure investment, which may shape community experiences of greenspace access, quality, safety, and health benefits. The review questions and Inclusion Criteria were revised to reflect this broadened scope across arid, semi-arid, temperate, tropical, urban, peri-urban, rural, and LMIC settings. |
| **Line 38 (major)**  *State what tool you will use to evaluate the grey literatures aside the JBI Critical Assessment tools. How would you manage the issue around using the method of the first 200 hits?* | Grey literature will now be appraised using the AACODS checklist (Authority, Accuracy, Coverage, Objectivity, Date, Significance), alongside the JBI Critical Appraisal Tools for empirical studies. This is stated in the abstract, Critical Appraisal, and Data Analysis sections. Two independent reviewers will complete each appraisal, with a third reviewer resolving disagreements; as with the JBI tools, results will inform interpretation rather than exclusion.  On reflection, we agreed the first-200-hits threshold could not be well justified. We removed the broad Google search entirely and instead will identify grey literature through ProQuest Dissertations and Theses Global, Google Scholar, and snowball searching of reference lists of included studies and key reviews—a more systematic and reproducible approach. |
| **Lines 39 and 165 (major)**  *Any plan for translation? The protocol does not specify how non-English studies will be translated or assessed for quality. This should also be mentioned briefly in the abstract.* | A translation plan was added to the Methods and briefly noted in the abstract, as requested. Non-English titles and abstracts will be translated using available translation tools and, when needed, reviewed by multilingual team members or qualified colleagues. Potentially eligible full texts will be translated using an interpreter or machine translation with human verification to safeguard translation quality. If an adequate translation cannot be obtained, the source will be documented and excluded with the reason reported. |
| **Line 248 (major)**  *Please clarify how you intend to adapt or apply GRADE appropriately to predominantly qualitative, and participatory evidences that would be gathered.* | We agree GRADE is not well suited to qualitative and participatory evidence. We replaced it with GRADE-CERQual, which was developed specifically to assess confidence in synthesized qualitative review findings; the abstract and Data Analysis section were revised accordingly. Mixed-methods or descriptive findings that do not fit GRADE-CERQual will be summarized narratively, informed by the JBI Critical Appraisal Tools and the AACODS checklist. |
| **Lines 326–328 (minor)**  *Reference 17 in the reference section seems missing in the entire body of the document. Please review the references and adjust as required.* | Thank you for catching this. Reference 17 (PRISMA) was cited at the end of the Studies/Source of Evidence Selection section but was not formatted like the other in-text citations, which is why it was not visible. We corrected the formatting, expanded the PRISMA and PRISMA-ScR acronyms at first mention, and reviewed all references for accuracy and consistency. |

**Additional Changes Made by the Authors**

| **Additional change** | **Description and rationale** |
| --- | --- |
| **Protocol registration** | Added the Open Science Framework registration (doi: 10.17605/OSF.IO/2DYGA) to the abstract and Introduction for transparency and comparability against the registered record. |
| **Finalized search strategy** | Rewrote the Search Strategy to reflect the finalized, librarian-developed approach: databases named in the abstract (PubMed, PsycINFO, Embase, ERIC, AMED, CINAHL, Web of Science) with platforms specified in the Methods; MEDLINE removed as redundant with PubMed; Appendix 1 replaced with the completed PubMed strategy (MeSH terms, keywords, retrieval counts) for reproducibility; PRISMA-ScR and PRISMA-S reporting extensions stated explicitly. |
| **Consistent terminology** | Standardized "greenspace" (one word) throughout the manuscript for consistency with the title and keywords. |
| **Refined review questions** | Reorganized the review questions to remove redundancy, group them under Population, Concepts, and Context, and align the Context questions with the broadened environmental scope; added a question on the unique contribution of photovoice to the greenspace-health literature. |
| **Clarified definitions and criteria** | Revised the opening definition of greenspace, added illustrative examples of health outcomes (e.g., community cohesion, improved mood), and removed the green stormwater infrastructure example to keep the greenspace typology consistent. |
| **Selection and appraisal procedures** | Clarified that citations will be exported to Covidence (now briefly described), disagreements resolved to consensus, and appraisals (JBI and AACODS) completed by two independent reviewers with a third reviewer available. |
| **Author information** | Corrected author names (Deborah Jean McClelland; Maiya Grace Block Ngaybe), added the omitted contribution statement for Huaqing Wang, credited D.J. McClelland’s search strategy development, and updated the Acknowledgments. |

We are grateful for the reviewer’s careful reading of the manuscript. The requested revisions, together with the additional changes above, have strengthened the protocol, and we hope the revised manuscript is now suitable for publication in PLOS ONE. We would be happy to make any further revisions the editorial team considers necessary.

Sincerely,

Rebecca Ramos

On behalf of all authors
