# Supplementary material for: A scoping review of photovoice studies concerning greenspaces and their impacts on health: Protocol
Source: PLoS One. 2026 Aug 4;21(8):e0355059. doi: 10.1371/journal.pone.0355059 (PMC13436740; doi:10.1371/journal.pone.0355059)
Supplement: Peer review history

# Peer review: Decision Letter 0

**DOI:** 10.1371/journal.pone.0355059.r001

**Authors:** Oluwaseun Badru

1 Jun 2026

PONE-D-26-17798

A Scoping Review of Photovoice Studies Concerning Greenspaces and Their Impacts on Health: Protocol

PLOS One

Dear Dr. Ramos,

Thank you for submitting your manuscript to PLOS ONE. After careful consideration, we feel that it has merit but does not fully meet PLOS ONE’s publication criteria as it currently stands. Therefore, we invite you to submit a revised version of the manuscript that addresses the points raised during the review process.

- **Photovoice is a great approach to understanding concepts, and it is worth exploring.**
- **Please address the comments from both reviewers.**

-->Please submit your revised manuscript by Jul 15 2026 11:59PM. If you will need more time than this to complete your revisions, please reply to this message or contact the journal office at plosone@plos.org. When you're ready to submit your revision, log on to https://www.editorialmanager.com/pone/ and select the 'Submissions Needing Revision' folder to locate your manuscript file.

Please include the following items when submitting your revised manuscript:

- A letter that responds to each point raised by the academic editor and reviewer(s). You should upload this letter as a separate file labeled 'Response to Reviewers'.
- A marked-up copy of your manuscript that highlights changes made to the original version. You should upload this as a separate file labeled 'Revised Manuscript with Track Changes'.
- An unmarked version of your revised paper without tracked changes. You should upload this as a separate file labeled 'Manuscript'.

If you would like to make changes to your financial disclosure, please include your updated statement in your cover letter. Guidelines for resubmitting your figure files are available below the reviewer comments at the end of this letter.

If applicable, we recommend that you deposit your laboratory protocols in protocols.io to enhance the reproducibility of your results. Protocols.io assigns your protocol its own identifier (DOI) so that it can be cited independently in the future. For instructions see: https://journals.plos.org/plosone/s/submission-guidelines#loc-laboratory-protocols. Additionally, PLOS ONE offers an option for publishing peer-reviewed Lab Protocol articles, which describe protocols hosted on protocols.io. Read more information on sharing protocols at https://plos.org/protocols?utm_medium=editorial-email&utm_source=authorletters&utm_campaign=protocols.

As the corresponding author, your ORCID iD is verified in the submission system and will appear in the published article. PLOS supports the use of ORCID, and we encourage all coauthors to register for an ORCID iD and use it as well. Please encourage your coauthors to verify their ORCID iD within the submission system before final acceptance, as unverified ORCID iDs will not appear in the published article. *Only*  the individual author can complete the verification step; PLOS staff *cannot*  verify ORCID iDs on behalf of authors.

We look forward to receiving your revised manuscript.

Kind regards,

Oluwaseun Abdulganiyu Badru,

Academic Editor

PLOS One

**Journal requirements:**

1. When submitting your revision, we need you to address these additional requirements.

Please ensure that your manuscript meets PLOS ONE's style requirements, including those for file naming. The PLOS ONE style templates can be found at

https://journals.plos.org/plosone/s/file?id=wjVg/PLOSOne_formatting_sample_main_body.pdf and

https://journals.plos.org/plosone/s/file?id=ba62/PLOSOne_formatting_sample_title_authors_affiliations.pdf

2. Thank you for stating in your Funding Statement:

Research reported in this publication was supported by the National Institute of Environmental Health Sciences of the National Institutes of Health under Award Number P20ES036112. The content is solely the responsibility of the authors and does not necessarily represent the official views of the National Institutes of Health.

Please provide an amended statement that declares *all* the funding or sources of support (whether external or internal to your organization) received during this study, as detailed online in our guide for authors at http://journals.plos.org/plosone/s/submit-now.  Please also include the statement “There was no additional external funding received for this study.” in your updated Funding Statement.

Please include your amended Funding Statement within your cover letter. We will change the online submission form on your behalf.

3. Thank you for stating the following financial disclosure:

Research reported in this publication was supported by the National Institute of Environmental Health Sciences of the National Institutes of Health under Award Number P20ES036112. The content is solely the responsibility of the authors and does not necessarily represent the official views of the National Institutes of Health.

Please state what role the funders took in the study.  If the funders had no role, please state: "The funders had no role in study design, data collection and analysis, decision to publish, or preparation of the manuscript."

If this statement is not correct you must amend it as needed.

Please include this amended Role of Funder statement in your cover letter; we will change the online submission form on your behalf.

[Note: HTML markup is below. Please do not edit.]

Reviewers' comments:

Reviewer's Responses to Questions

**Comments to the Author**

1. Does the manuscript provide a valid rationale for the proposed study, with clearly identified and justified research questions?

The research question outlined is expected to address a valid academic problem or topic and contribute to the base of knowledge in the field.

Reviewer #1: Yes

Reviewer #2: Yes

**********

2. Is the protocol technically sound and planned in a manner that will lead to a meaningful outcome and allow testing the stated hypotheses?

The manuscript should describe the methods in sufficient detail to prevent undisclosed flexibility in the experimental procedure or analysis pipeline, including sufficient outcome-neutral conditions (e.g. necessary controls, absence of floor or ceiling effects) to test the proposed hypotheses and a statistical power analysis where applicable. As there may be aspects of the methodology and analysis which can only be refined once the work is undertaken, authors should outline potential assumptions and explicitly describe what aspects of the proposed analyses, if any, are exploratory.

Reviewer #1: Yes

Reviewer #2: Yes

**********

3. Is the methodology feasible and described in sufficient detail to allow the work to be replicable?

Descriptions of methods and materials in the protocol should be reported in sufficient detail for another researcher to reproduce all experiments and analyses. The protocol should describe the appropriate controls, sample size calculations, and replication needed to ensure that the data are robust and reproducible.

Reviewer #1: Yes

Reviewer #2: Yes

**********

4. Have the authors described where all data underlying the findings will be made available when the study is complete?

The PLOS Data policy requires authors to make all data underlying the findings described in their manuscript fully available without restriction, with rare exception, at the time of publication. The data should be provided as part of the manuscript or its supporting information, or deposited to a public repository. For example, in addition to summary statistics, the data points behind means, medians and variance measures should be available. If there are restrictions on publicly sharing data—e.g. participant privacy or use of data from a third party—those must be specified.

Reviewer #1: Yes

Reviewer #2: Yes

**********

5. Is the manuscript presented in an intelligible fashion and written in standard English?

PLOS ONE does not copyedit accepted manuscripts, so the language in submitted articles must be clear, correct, and unambiguous. Any typographical or grammatical errors should be corrected at revision, so please note any specific errors here.

Reviewer #1: Yes

Reviewer #2: Yes

**********

6. Review Comments to the Author

Please use the space provided to explain your answers to the questions above and, if applicable, provide comments about issues authors must address before this protocol can be accepted for publication. You may also include additional comments for the author, including concerns about research or publication ethics.

You may also provide optional suggestions and comments to authors that they might find helpful in planning their study.

(Please upload your review as an attachment if it exceeds 20,000 characters)

**Reviewer #1:**  27- 28

1. This statement creates some ambiguity to me. What is the definition of underrepresented environments?

2. Regarding the examples of the underrepresented environments’ given in the protocol, arid was more spoken about more whereas as stated in line 132-134 that ‘No restrictions will be applied regarding geographic location, setting (urban, Peri-urban, or rural), or population’. The way the document is written (like in line 63), presents review as majorly concerned with arid regions but not the other regions that fall under tropical, temperate, continental or polar climate.

Line 38

1. Since grey literature documents are rarely peer-reviewed how will you assess their quality and reliability? What tool will you be using to evaluate the Authority, Accuracy, Coverage, Objectivity, Date, and Significance of the grey literature?

2. The decision to limit the broad Google search to the first 200 hits (line 202), although pragmatic, may benefit from a brief justification, as this threshold could miss relevant community or organizational reports.

Lines 39 and 165

Any plan for translation? The protocol does not specify how non-English studies will be translated or assessed for quality, which is a practical gap worth addressing.

As stated in line 165, there is a need to state how you would manage issues with findings that are not presented in English, and how the quality of the translations. This should also be mentioned briefly in the abstract.

Line 248

The use of GRADE, typically designed for intervention-based evidence, especially in healthcare is noted. Could you please clarify how you intend to adapt or apply GRADE appropriately to predominantly qualitative, and participatory evidences that would be gathered.

Lines 326-328

Reference 17 in the reference section seems missing in the entire body of the document. Please revise and update as required.

Kindly see the attached word document for more details.

**Reviewer #2:**  Thank you for the opportunity to review this protocol. While the protocol is well-written, I have identified some issues and provided recommendations to further improve its quality.

1. Title: Although it is grammatically correct. I suggest revising the title to improve clarity.

2. Since the study focuses on specific environments, I suggest incorporating location in the title. This information will help prepare the readers for what to expect from the study.

Abstract

3. The phrase “To identify community actions and advocacy arising from these participatory projects” is somewhat unclear in terms of how these concepts will be defined. It is also uncertain whether the review aims to describe, categorize, or evaluate such outcomes. Additionally, there is no connection established between these actions and the use of photovoice, and the introduction lacks further details on this matter. I recommend clarifying this language and ensuring that it aligns consistently with the objectives of the

4. The method section did not adequately summarize the methodological rigor described in the body of the manuscript and omitted information on how disagreements would be resolved and how results would be presented.

Introduction

5. There is inconsistency in the use of the terms "greenspaces" and "green spaces.

6. Green spaces are defined as publicly accessible land covered with natural vegetation. This definition appears to be restrictive and may not adequately reflect other environments discussed later in the manuscript, such as green infrastructure and managed landscapes (e.g., golf courses). This suggests that green spaces may not always be publicly accessible and can vary in their levels of naturalness. I recommend refining the definition to encompass a broader range of concepts.

7. In lines 53-57, the transition between discussing the health benefits of exposure to green spaces and critiquing predominantly quantitative research feels somewhat abrupt and would benefit from improved clarity. Also, the introduction of photovoice in this section comes across more as a justification for using this method in primary research rather than a synthesis of existing photovoice studies. It would be helpful to clearly frame photovoice within the context of the review’s purpose, specifically addressing why it is important to systematically map and synthesize this body of evidence.

8. Line 58 -59: The opening statement is somewhat repetitive since earlier sections already established that extensive studies have been conducted. Although it introduces the idea of growing interest, it does not clearly contribute to the rationale. I recommend strengthening the transition to the identified gap and adding supporting references to bolster these claims within the paragraph.

9. Line 59 - 82: This paragraph introduces several gaps without clear prioritization, making it difficult to identify the core justification of the study. While the rationale discusses geographic disparities (high-income countries vs. low- and middle-income countries), it is not well integrated with the focus on photovoice and environmental context. The discussion of arid vs. non-arid contexts is somewhat repetitive. I suggest clearly emphasizing the main gap driving the review and ensuring that supporting points are tightly linked to this central rationale

10. The study primarily focuses on synthesizing photovoice; however, the introduction emphasizes green spaces more than photovoice. I recommend expanding the discussion of photovoice, specifically how and why it has been used to study green spaces and community health, and more clearly positioning it as the central focus of the review.

11. A comparative understanding of impact suggests a focus on effect comparison, which does not align with the study's aim. The study is focused on mapping perceptions and types of evidence, not quantifying effects.

12. Line 83 – 98: I recommend linking the rationale for selecting a scoping review more specifically to the particular gap the study addresses.

13. Line 99 – 107: There are redundancies between this paragraph and the previous one. I suggest revising for conciseness. Although the review focuses on photovoice studies, the objectives do not clearly reflect this, reading more like a green space review instead. The objective is too dense and combines multiple aims into a single sentence. I suggest making it more concise.

14. Inclusion criteria: It states that the review will include participants of any age. Does this include children as well? You may want to clarify this point..

Concept

15. Information regarding inclusion and exclusion criteria is mixed with the main concept. I recommend separating these details into the inclusion section.

16. I suggest briefly explaining what "green space" refers to here rather than directing readers back to the introduction

17. Excluding studies that address distal or systemic outcomes may limit the evidence directly relevant to identifying community actions and advocacy stemming from photovoice projects. I recommend reviewing how these outcomes will be managed.

18. The section on types of sources largely repeats information from the inclusion and concept sections. I suggest structuring this section according to the Population–Concept–Context framework to provide a clearer justification for exclusions and better alignment with JBI scoping review methodology.

19. Please clarify this statement: “Systematic reviews will be excluded unless they contain primary data not otherwise available in published studies,” as systematic reviews typically do not present primary data.

Method

20. There is a mix of past and future tenses throughout the document. I recommend addressing these grammatical inconsistencies.

21. I suggest providing a justification for the use of human filters.

22. Although the search end date is mentioned in the abstract, it should also be included in the main methods section

23. I recommend ensuring that studies using visual participatory approaches are not excluded simply because the term "photovoice" does not appear in their titles, even if the method is used.

24. Since non-English studies will be included, I suggest explaining how translations will be handled.

25. Please describe what the pilot test entails

26. You should detail how disagreements between reviewers will be resolved beyond just discussion.

27. Line 214 -215: Does this mean that each reviewer will create their own individual data abstraction form?

28. I recommend adding information on how inter-rater reliability will be assessed.

29. Please provide details on which thematic synthesis approach will be utilized, how coding will be conducted, and how themes will be validated.

30. Photovoice studies employ diverse methods—specifically in terms of population, outcomes, purpose, and context. It would be helpful to explain how these variations will be addressed.

31. You may also want to explain how the appraisal result will be presented

32. GRADE is primarily used for evaluating intervention effectiveness and ascertaining evidence. It may not be suitable for identifying research gaps, variations in geographic or environmental contexts, and implications for future participatory research, which is the focus of this review. I recommend reviewing this.

33. Mapping techniques, such as conceptual diagrams or frameworks, may be employed to visually represent the relationships among photovoice methodologies, greenspace contexts, and health impacts identified across studies. The use of the word "may" implies that this has not been finalized; I suggest revising this statement to confirm which techniques will be utilized.

**********

7. PLOS authors have the option to publish the peer review history of their article (what does this mean?). If published, this will include your full peer review and any attached files.

If you choose “no”, your identity will remain anonymous but your review may still be made public.

**Do you want your identity to be public for this peer review?**  For information about this choice, including consent withdrawal, please see our Privacy Policy.

Reviewer #1: **Yes:**  Bolaji Mathew, Akala

Reviewer #2: **Yes:**  Mojirade Adetoba

**********

[NOTE: If reviewer comments were submitted as an attachment file, they will be attached to this email and accessible via the submission site. Please log into your account, locate the manuscript record, and check for the action link "View Attachments". If this link does not appear, there are no attachment files.]

To ensure your figures meet our technical requirements, please review our figure guidelines: https://journals.plos.org/plosone/s/figures

You may also use PLOS’s free figure tool, NAAS, to help you prepare publication quality figures: https://journals.plos.org/plosone/s/figures#loc-tools-for-figure-preparation.

NAAS will assess whether your figures meet our technical requirements by comparing each figure against our figure specifications.

Attachment Submitted filename: PLOS One-A Scoping Review (BA-22ndMay, 2026).docx

# Author comment: Author response to Decision Letter 1

**DOI:** 10.1371/journal.pone.0355059.r002

15 Jul 2026

I have attached the file responding to all reviewers. Thank you!

Attachment Submitted filename: Response to Editor_20260713.docx

# Editor report: Decision Letter 1

**DOI:** 10.1371/journal.pone.0355059.r003

**Authors:** Oluwaseun Badru

17 Jul 2026

A Scoping Review of Photovoice Studies Concerning Greenspaces and Their Impacts on Health: Protocol

PONE-D-26-17798R1

Dear Dr. Ramos,

We’re pleased to inform you that your manuscript has been judged scientifically suitable for publication and will be formally accepted for publication once it meets all outstanding technical requirements.

Within one week, you’ll receive an e-mail detailing the required amendments. When these have been addressed, you’ll receive a formal acceptance letter and your manuscript will be scheduled for publication.

An invoice will be generated when your article is formally accepted. Please note, if your institution has a publishing partnership with PLOS and your article meets the relevant criteria, all or part of your publication costs will be covered. Please make sure your user information is up-to-date by logging into Editorial Manager at Editorial Manager® and clicking the ‘Update My Information' link at the top of the page. For questions related to billing, please contact billing support.

If your institution or institutions have a press office, please notify them about your upcoming paper to help maximize its impact. If they’ll be preparing press materials, please inform our press team as soon as possible -- no later than 48 hours after receiving the formal acceptance. Your manuscript will remain under strict press embargo until 2 pm Eastern Time on the date of publication. For more information, please contact onepress@plos.org.

Kind regards,

Oluwaseun Abdulganiyu Badru,

Academic Editor

PLOS One

# Editor report: Acceptance letter

**DOI:** 10.1371/journal.pone.0355059.r004

**Authors:** Oluwaseun Badru

PONE-D-26-17798R1

PLOS One

Dear Dr. Ramos,

I'm pleased to inform you that your manuscript has been deemed suitable for publication in PLOS One. Congratulations! Your manuscript is now being handed over to our production team.

At this stage, our production department will prepare your paper for publication. This includes ensuring the following:

* All references, tables, and figures are properly cited

* All relevant supporting information is included in the manuscript submission,

* There are no issues that prevent the paper from being properly typeset

You will receive further instructions from the production team, including instructions on how to review your proof when it is ready. Please keep in mind that we are working through a large volume of accepted articles, so please give us a few days to review your paper and let you know the next and final steps.

Lastly, if your institution or institutions have a press office, please let them know about your upcoming paper now to help maximize its impact. If they'll be preparing press materials, please inform our press team within the next 48 hours. Your manuscript will remain under strict press embargo until 2 pm Eastern Time on the date of publication. For more information, please contact onepress@plos.org.

You will receive an invoice from PLOS for your publication fee after your manuscript has reached the completed accept phase. If you receive an email requesting payment before acceptance or for any other service, this may be a phishing scheme. Learn how to identify phishing emails and protect your accounts at https://explore.plos.org/phishing.

If we can help with anything else, please email us at customercare@plos.org.

Thank you for submitting your work to PLOS ONE and supporting open access.

Kind regards,

PLOS ONE Editorial Office Staff

on behalf of

Dr. Oluwaseun Abdulganiyu Badru

Academic Editor

PLOS One
