# Supplementary material for: Correction: A scoping review of photovoice studies concerning greenspaces and their impacts on health: Protocol
Source: PLoS One. 2026 Oct 23;21(10):e0361454. doi: 10.1371/journal.pone.0361454 (PMC13693944; doi:10.1371/journal.pone.0361454)
Supplement: Supplementary file 1 [file pone.0361454.s001.pdf]

PLOS One

STUDY PROTOCOL

# A scoping review of photovoice studies concerning greenspaces and their impacts on health: Protocol

**Chidera M. Ejike[1], Rebecca Ramos[2], Christiana Ketema[3], Mackenzie Waller[4], Kai Glahn[5], Auwal Abubakar[6], Jean McClelland[7], Kacey Ernst[1], Huaqing Wang[8], Shujuan Li[4], Maiya G. Block Ngaybe[4]***

**1** Department of Epidemiology and Biostatistics, The University of Arizona, Tucson, Arizona, United States of America, **2** Department of Community, Environment, and Policy, The University of Arizona, Tucson, Arizona, United States of America, **3** Mel and Enid Zuckerman College of Public Health, The University of Arizona, Tucson, Arizona, United States of America, **4** College of Architectural Planning and Landscape Architecture, The University of Arizona, Tucson, Arizona, United States of America, **5** Department of Psychiatry, The University of Arizona, Tucson, Arizona, United States of America, **6** The University of Arizona, Tucson, Arizona, United States of America, **7** Arizona Health Sciences Library, The University of Arizona, Tucson, Arizona, United States of America, **8** Department of Landscape Architecture and Environmental Planning, Utah State University, Logan, Utah, United States of America

* rebeccaramos08@arizona.edu

OPEN ACCESS

**Citation:** Ejike CM, Ramos R, Ketema C, Waller M, Glahn K, Abubakar A, et al. (2026) A scoping review of photovoice studies concerning greenspaces and their impacts on health: Protocol. PLoS One 21(8): e0355059. https://doi.org/10.1371/journal.pone.0355059

**Editor:** Oluwaseun Abdulganiyu Badru, The University of Iowa College of Public Health, UNITED STATES OF AMERICA

**Received:** April 29, 2026

**Accepted:** July 16, 2026

**Published:** August 4, 2026

**Peer Review History:** PLOS recognizes the benefits of transparency in the peer review process; therefore, we enable the publication of all of the content of peer review and author responses alongside final, published articles. The editorial history of this article is available here: https://doi.org/10.1371/journal.pone.0355059

**Copyright:** © 2026 Ejike et al. This is an open access article distributed under the terms of the Creative Commons Attribution License, which permits unrestricted use, distribution,

## Abstract

### Objective

The objective of this scoping review is to characterize the extent and nature of the available research on photovoice studies examining how greenspaces influence community health, and to identify community actions and advocacy arising from these participatory projects.

### Introduction

Greenspaces have been shown to support physical, mental, and social health. Yet research often overlooks community perspectives, *particularly in underrepresented environments such as arid regions*. Photovoice enables individuals to document and reflect on their lived environments, providing unique insights into the relationships between greenspaces, health, and community wellbeing. Synthesizing photovoice studies that examine how greenspaces impact health across different ecosystems and landscape designs is essential to capture community-driven knowledge and outcomes, including initiatives that inform policy and practice.

### Inclusion criteria

This review will include primary studies employing photovoice methods with human participants, of any demographic or cultural background, that focus on greenspaces and associated health outcomes. Eligible studies may use qualitative or

and reproduction in any medium, provided the original author and source are credited.

**Data availability statement:** N/A - No results are reported.

**Funding:** Research reported in this publication was supported by the National Institute of Environmental Health Sciences of the National Institutes of Health under Award Number P20ES036112. The content is solely the responsibility of the authors and does not necessarily represent the official views of the National Institutes of Health.

**Competing interests:** The authors have declared that no competing interests exist.

mixed-methods designs, provided photovoice is a core component. Quantitative-only studies, reviews, editorials, and those addressing distal health measures will be excluded.

## Methods

Searches will be conducted in electronic databases and relevant grey literature sources. No language restrictions will be applied, and the time frame will extend from January 1997, when photovoice was first described, to the present. Screening and data extraction will be conducted in duplicate using Covidence. Findings will be summarized descriptively and synthesized thematically, and confidence in key review findings will be assessed using the JBI Critical Assessment Tools.

## Introduction

Greenspaces (typically defined as publicly accessible land, primarily covered with natural vegetation such as grass, trees, and plants) play a vital role in urban and rural environments. These spaces include parks, gardens, tree-lined streets, planted lots, and urban forests [1]. Extensive research has demonstrated that green spaces offer numerous benefits to human health, including physical, mental, social, and emotional well-being [2,3]. Exposure to greenspaces is associated with reductions in stress, improvements in cardiovascular health, increases in physical activity, and enhancements in community cohesion and social capital [4,5]. However, much of the existing literature focuses on quantitative outcomes and often overlooks community members' lived experiences and perceptions of how green spaces impact their health. Participatory research methods, such as photovoice, offer a unique avenue for exploring and understanding community perspectives on the relationship between greenspaces and health [6].

There is a growing academic and policy interest in understanding how greenspaces impact health outcomes. Yet, despite this interest, there is a notable gap in synthesizing evidence, specifically from photovoice studies, that captures community voices and actions related to greenspaces and health. These participatory studies often reveal nuanced influences of greenspace quality, accessibility, cultural significance, and environmental justice aspects on perceived health benefits or harms [7]. Furthermore, research focused on greenspaces in arid environments is sparse [8]. Environments such as arid versus non-arid regions present distinct challenges and experiences regarding green space characteristics and how they may impact health. Greenspaces in arid environments may differ significantly in vegetation, water usage, and community engagement when compared to more temperate settings, for example [7,8]. These ecological differences translate into distinct community experiences and challenges. For instance, arid green spaces may require resource adaptations, such as prioritizing drought-tolerant native plants, and face competing demands between ecological sustainability and community desires for lush vegetation [9]. Evidence on how these landscape qualities impact health and well-being, particularly

from the perspective of residents in arid regions, is sparse and fragmented, with studies dispersed across disciplines and employing varied methodologies. Thus, arid communities' unique environmental strains coupled with their localized priorities create complex dynamics in green space accessibility, use, and perceived health impacts that have yet to be comprehensively studied [10]. A comparative understanding of the impacts of arid and non-arid greenspaces on health would help tailor equitable and ecologically sound strategies for public health and urban planning [11].

Studies examining how greenspaces impact community health are more prevalent in High Income Countries (HICs), such as the Netherlands, China, and the United States [12]. However, there is a need for studies in Low- and Middle-Income Countries (LMICs). Therefore, examining where this research is conducted globally is important for identifying gaps and understanding differences in cultural contexts, communities' lived experiences, and local priorities [12,13].

A scoping review design was selected as the most appropriate methodology for several reasons. Unlike systematic reviews, which focus on more specific questions and often assess the effectiveness of interventions, scoping reviews are ideal for mapping broader, more complex, and emerging fields of evidence [14]. This review's objective to explore participatory methodologies, diverse health outcomes, and various environmental contexts aligns well with the exploratory and descriptive nature of scoping reviews. Additionally, as photovoice studies employ heterogeneous methods and report a wide range of qualitative data, a scoping review can comprehensively capture this variation, laying the groundwork for future targeted systematic assessments or qualitative syntheses [15]. A preliminary search of MEDLINE, the Cochrane Database of Systematic Reviews, and the JBI Evidence Synthesis database did not identify any current or ongoing scoping reviews specifically addressing photovoice studies on greenspaces and their impacts on community health. This review will therefore fill a critical gap by clarifying how different types of green spaces are valued and utilized across diverse settings, illuminating the breadth of health outcomes identified by communities, and providing an organized synthesis accessible to researchers, policymakers, public health practitioners, and community stakeholders aiming to leverage community-engaged research for health-promoting green space interventions.

The objective of this scoping review is to map and synthesize the available evidence on photovoice studies examining the impacts of green spaces on community health and community actions that arise from these participatory projects, with particular attention to environmental context differences, including arid and non-arid settings. This review will address the following gaps: (1) the range and types of green spaces studied through photovoice (with an emphasis on arid versus non-arid settings); (2) the health outcomes identified through community perspectives; and (3) the community-driven advocacy, policy influence, or planning that emerges as a result of photovoice research. This review will adhere to the Johanna Briggs Institute (JBI) methodology [16] to ensure comprehensive and transparent reporting of findings.

## Review questions

### *Population*

- What are the characteristics of the communities participating in photovoice greenspace studies (e.g., geographic location, socio-demographics, etc.)?

### *Concepts*

- How are photovoice studies used to examine the relationship between green spaces and community health?
- What types of green spaces are examined in photovoice studies?
- What health outcomes related to green space exposure are identified in photovoice studies?
- How is greenspace defined across photovoice studies?

- What forms of community action or advocacy emerge from photovoice studies on green spaces and health?
- What ethical considerations are addressed in photovoice studies, and are any unique to research on greenspaces?

### *Context*

- Do community perceptions of greenspace health differ between arid and non-arid environments?
- In which geographic or environmental contexts (urban/rural, arid/non-arid) are the studies being conducted?
- How do contextual differences influence health outcomes related to green spaces?

## Inclusion criteria

### *Participants*

This review will include studies involving human participants of any age, gender, socioeconomic status, and/or cultural background. Eligible participants may be individuals, groups, or communities engaged in photovoice projects that explore green spaces and health. No restrictions will be applied regarding geographic location, setting (urban, peri-urban, or rural), or population type. Studies will be excluded if participants are not directly engaged in photovoice activities (e.g., projects in which photographs are taken or analyzed solely by researchers without community involvement), unless the term photovoice was explicitly mentioned as the methodology used in such studies.

### *Concept*

The central concept of this scoping review is the application of the photovoice methodology to investigate how community members perceive and experience greenspaces and their impacts on health and well-being. Photovoice is a participatory research approach in which community members use photography, combined with critical dialogue, to document, reflect on, and communicate their lived experiences within their environment [6]. Eligible studies must explicitly employ photovoice as a core design, where participants take photographs reflecting their lived experiences and engage in participatory analysis and dissemination with researchers. Quantitative-only studies, reviews, editorials, and other non-original research will be excluded, while mixed-methods studies will be included only when photovoice is a distinct, reported component. For the purposes of this review, studies must clearly investigate greenspaces as defined in the introductory section; those that broadly address the "built environment" without distinct exposure to greenspace will be excluded unless greenspace findings can be extracted and analyzed separately. Health outcomes of interest include direct individual-level impacts of greenspace exposure, such as physical (e.g., cardiovascular health), mental (e.g., stress reduction, mental wellbeing), social, and emotional outcomes. Studies that focus exclusively on distal or systemic outcomes (e.g., social determinants of health or policy discussions without direct measurement of health outcomes) will be excluded. Where available, this review will also chart differences in health impacts across types of greenspaces, including managed environments (e.g., maintained parks, golf courses) and naturalistic or native-plant landscapes such as green stormwater infrastructure.

### *Context*

This review will consider studies conducted in any geographic or cultural setting where photovoice has been applied to explore greenspaces and health. Eligible contexts include urban, peri-urban, and rural environments, across diverse climates, including arid, semi-arid, temperate, and tropical regions. Studies may be situated in community, educational, residential, or clinical settings, provided the focus remains on greenspaces as defined in the Concept section. No restrictions will be placed on country, region, or income level, ensuring global perspectives are captured. Similarly, there will be no language restrictions; studies published in languages other than English will be included if sufficient information can be

obtained through translation. Exclusions will apply to studies examining environments not characterized as green spaces. (e.g., purely built environments without vegetation). Studies conducted exclusively in virtual or simulated environments will also be excluded.

### Types of sources

This scoping review will primarily consider qualitative studies, as photovoice is a participatory qualitative methodology. We will include primary research articles in which photovoice is the main methodology or is part of a mixed-methods study. Quantitative studies, narrative reviews, editorials, commentaries, and opinion papers without a distinct photovoice component will be excluded. In addition, Systematic reviews will be excluded unless they contain primary data not otherwise available in published studies. Grey literature sources such as dissertations, theses, community or organizational reports, and other non–peer-reviewed outputs will be considered where they report original photovoice findings.

## Methods

The proposed scoping review will be conducted in accordance with the JBI methodology [16].

### *Search strategy*

The search strategy aims to identify both published and unpublished studies. A three-step search approach will be used in this review. First, a brief initial search of PubMed, MEDLINE, PsycINFO, ERIC, AMED, CINAHL, Embase, ProQuest Dissertations and Theses Global, and Web of Science was conducted to find articles on Photovoice methodology, Greenspace exposure, and health outcomes. The keywords identified in the titles and abstracts of relevant articles, along with the index terms used to describe them, were used to develop a comprehensive search strategy to identify relevant databases and sources (see S1 File Appendix 1). This search strategy, including all identified keywords and index terms, will be tailored for each database and source included. When possible, the human filter will be applied.

Studies published in any language will be included to highlight disparities by language or by underrepresented regions worldwide in this research area. Studies published from 1997 onward will be included, as the original articles describing the photovoice methodology appeared in the late 1990s. The search strategies will be developed with input from the project team and refined in consultation with an experienced health sciences librarian familiar with scoping review methodology. The initial comprehensive PubMed search strategy will be created and then adapted for other databases, taking into account their controlled vocabularies and syntax. Snowball sampling techniques, including screening reference lists of included studies and key reviews, will be used to identify additional relevant sources. The electronic databases to be searched include PubMed, MEDLINE, PsycINFO, ERIC, AMED, CINAHL, Embase, ProQuest Dissertations and Theses Global, and Web of Science. Sources of unpublished studies and grey literature to be searched include Google Scholar and a broad Google search of the first 200 hits to scan relevant websites.

### Studies/source of evidence selection

After the initial search, all identified citations will be compiled and uploaded to Covidence, and duplicates will be eliminated. Following a pilot test, titles and abstracts will be screened by two or more independent reviewers to evaluate compliance with the review's inclusion criteria. Reasons for excluding evidence sources at the full-text level that do not meet the inclusion criteria will be recorded and reported in the scoping review. Any disagreements between reviewers at each stage of the selection process will be resolved through discussion or by involving additional reviewers. The search results and the study inclusion process will be fully reported in the final scoping review, presented in a PRISMA flow diagram, and documented according to PRISMA-P (S2 File) [17].

### Data extraction

Data will be extracted from papers included in the scoping review by two or more independent reviewers using a data extraction tool developed by the reviewers. The extracted data will include specific details on the participants, the concept, the context, the study methods, and key findings relevant to the review question(s). A draft extraction form is provided (see S1 File Appendix 2). The draft data extraction tool will be revised as necessary during the data extraction process for each included evidence source. Main outcomes extracted will be physical, mental, and social/community health because they are most central to the review questions, while additional outcomes will include environmental, behavioral, advocacy, policy, access, and other emergent community-defined impacts to capture the full scope of evidence. Modifications will be detailed in the scoping review. Any disagreements between the reviewers will be resolved through discussion or by involving additional reviewers. If appropriate, authors of papers will be contacted to request missing or additional data, where required.

### Critical appraisal of individual sources of evidence

To provide additional context regarding the methodological rigor of the included photovoice studies, a quality assessment will be performed using the Johanna Briggs Institute (JBI) Critical Appraisal Tools. The JBI critical appraisal tools are well-suited for evaluating qualitative research, focusing on study validity, methodology, ethical considerations, data analysis, and relevance of findings. This appraisal will not be used to exclude studies but rather to inform the interpretation and synthesis of evidence, identify gaps related to study quality, and guide recommendations for future research. Two independent reviewers will apply the JBI critical appraisal checklists to each included study, with disagreements resolved through discussion or consultation with a third reviewer.

### Data analysis and presentation

The evidence gathered from this review will be systematically organized and presented to directly address the review objective and research questions. Quantitative descriptive data, such as study characteristics, participant demographics, and types of green spaces studied, will be summarized in tabular form to provide a clear overview of the existing literature. Qualitative findings, including community perceptions of greenspaces, reported health outcomes, and community-driven actions, will be synthesized thematically. Mapping techniques such as conceptual diagrams or frameworks may be used to visually represent relationships among photovoice methodologies, greenspace contexts, and health impacts identified across studies. A narrative summary will accompany all tabulated and/or graphical data, provide interpretative context, and explicitly link the results to the review's objectives and specific review questions. This approach will support the identification of research gaps, variations by geographic or environmental context, and implications for future participatory research. The strength of the body of evidence will be assessed using the GRADE (Grading of Recommendations, Assessment, Development, and Evaluations) approach.

## Supporting information

**S1 File. Appendices.** Appendix 1: PubMed Search Strategy & Appendix 2: Data Extraction Instrument.
(DOCX)

**S2 File. PRISMA-P Checklist.** PRISMA-P (Preferred Reporting Items for Systematic review and Meta-Analysis Protocols) 2015 checklist.
(DOCX)

## Acknowledgments

A special thank you to Shujuan Li, Kacey Ernst, and Noah Ortega for their support on this project.

## Author contributions

**Conceptualization:** Chidera M Ejike, Maiya G Block Ngaybe.

**Funding acquisition:** Shujuan Li.

**Investigation:** Chidera M Ejike, Maiya G Block Ngaybe.

**Methodology:** Chidera M Ejike, Maiya G Block Ngaybe.

**Supervision:** Maiya G Block Ngaybe.

**Writing – original draft:** Rebecca Ramos, Chidera M Ejike, Christiana Ketema, Maiya G Block Ngaybe.

**Writing – review & editing:** Rebecca Ramos, Chidera M Ejike, Christiana Ketema, Mackenzie Waller, Kai Glahn, Auwal Abubakar, Jean McClelland, Kacey Ernst, Huaqing Wang, Shujuan Li, Maiya G Block Ngaybe.

## References


1. Twohig-Bennett C, Jones A. The health benefits of the great outdoors: a systematic review and meta-analysis of greenspace exposure and health outcomes. Environ Res. 2018;166:628–37. https://doi.org/10.1016/j.envres.2018.06.030 PMID: 29982151
2. Hartig T, Mitchell R, de Vries S, Frumkin H. Nature and health. Annu Rev Public Health. 2014;35:207–28.
3. Lee ACK, Maheswaran R. The health benefits of urban green spaces: a review of the evidence. J Public Health (Oxf). 2011;33(2):212–22. https://doi.org/10.1093/pubmed/fdq068 PMID: 20833671
4. Callaghan A, McCombe G, Harrold A, McMeel C, Mills G, Moore-Cherry N, et al. The impact of green spaces on mental health in urban settings: a scoping review. J Ment Health. 2021;30(2):179–93. https://doi.org/10.1080/09638237.2020.1755027 PMID: 32310728
5. Dzhambov AM, Dimitrova DD, Dimitrakova ED. Association between residential greenness and birth weight: systematic review and meta-analysis. Urban Forest Urban Green. 2014;13(4):621–9. https://doi.org/10.1016/j.ufug.2014.09.004
6. Wang C, Burris MA. Photovoice: concept, methodology, and use for participatory needs assessment. Health Educ Behav. 1997;24(3):369–87.
7. Mitchell RJ, Richardson EA, Shortt NK, Pearce JR. Neighborhood environments and socioeconomic inequalities in mental well-being. Am J Prev Med. 2015;49(1):80–4. https://doi.org/10.1016/j.amepre.2015.01.017 PMID: 25911270
8. Bowler DE, Buyung-Ali LM, Knight TM, Pullin AS. A systematic review of evidence for the added benefits to health of exposure to natural environments. BMC Public Health. 2010;10:456. https://doi.org/10.1186/1471-2458-10-456 PMID: 20684754
9. (PDF) A spatial vulnerability analysis of urban populations during extreme heat events in Australian capital cities Final Report [Internet]. ResearchGate. [cited 2025 Sept 25]. https://www.researchgate.net/publication/235725298_A_spatial_vulnerability_analysis_of_urban_populations_during_extreme_heat_events_in_Australian_capital_cities_Final_Report
10. Norton BA, Coutts AM, Livesley SJ, Harris RJ, Hunter AM, Williams NSG. Planning for cooler cities: a framework to prioritise green infrastructure to mitigate high temperatures in urban landscapes. Landscape and Urban Planning. 2015;134:127–38. https://doi.org/10.1016/j.landurbplan.2014.10.018
11. Rigolon A, Browning MHEM, McAnirlin O, Yoon H. Green space and health equity: a systematic review on the potential of green space to reduce health disparities. Int J Environ Res Public Health. 2021 Mar 4;18(5):2563.
12. Shuvo FK, Feng X, Akaraci S, Astell-Burt T. Urban green space and health in low and middle-income countries: a critical review. Urban Forest Urban Green. 2020;52:126662. https://doi.org/10.1016/j.ufug.2020.126662
13. Xian Z, Nakaya T, Liu K, Zhao B, Zhang J, Zhang J, et al. The effects of neighbourhood green spaces on mental health of disadvantaged groups: a systematic review. Humanit Soc Sci Commun. 2024 Apr 5;11(1):488.
14. Peters MDJ, Godfrey C, McInerney P, Khalil H, Larsen P, Marnie C, et al. Best practice guidance and reporting items for the development of scoping review protocols. JBI Evid Synth. 2022;20(4):953–68. https://doi.org/10.11124/JBIES-21-00242 PMID: 35102103
15. Levac D, Colquhoun H, O'Brien KK. Scoping studies: advancing the methodology. Implement Sci. 2010;5:69. https://doi.org/10.1186/1748-5908-5-69 PMID: 20854677
16. Aromataris E, Lockwood C, Porritt K, Pilla B, Jordan Z. JBI Manual for Evidence Synthesis. https://synthesismanual.jbi.global
17. Page MJ, McKenzie JE, Bossuyt PM, Boutron I, Hoffmann TC, Mulrow CD, et al. The PRISMA 2020 statement: an updated guideline for reporting systematic reviews. 2021. https://www.bmj.com/content/372/bmj.n71
